# Role of LncRNA MRPS28 in Secondary Hair Follicle Development of Cashmere Goats

**DOI:** 10.3390/ani15131882

**Published:** 2025-06-25

**Authors:** Youjun Rong, Rong Ma, Qing Ma, Bingjie Ma, Xuxu Bao, Yiming Zhang, Le Wang, Fangzheng Shang, Ruijun Wang, Rui Su, Yu Wang, Yanjun Zhang

**Affiliations:** 1 College of Animal Science, Inner Mongolia Agricultural University, Hohhot 010018, China; rongyoujun1@163.com (Y.R.);; 2 College of Veterinary Medicine, Inner Mongolia Agricultural University, Hohhot 010018, China; 3 Key Laboratory of Mutton Sheep Genetics and Breeding, Ministry of Agriculture, Hohhot 010018, China; 4 Key Laboratory of Goat and Sheep Genetics, Breeding and Reproduction in Inner Mongolia Autonomous Region, Hohhot 010018, China

**Keywords:** cashmere goats, secondary hair follicle, ceRNA, lncRNA MRPS28, NUAK1

## Abstract

The development of secondary hair follicles in cashmere goats directly influences the yield and quality of cashmere, and hair follicle morphogenesis is a complex biological process involving multiple signaling pathways and regulatory factors. In recent years, the regulatory role of long non-coding RNAs in hair follicle development has gradually been uncovered. Based on a previously established transcriptome database of embryonic skin tissue from cashmere goats, this study ultimately identified lncRNA MRPS28, which exhibits significant differential expression during the morphogenesis of secondary hair follicles. Our results indicate that lncRNA MRPS28 is a non-coding RNA transcribed from the intron region of the *MRPS28* gene. Additionally, it can inhibit the proliferation and migration of dermal fibroblasts while promoting their apoptosis. Further mechanistic studies have shown that lncRNA MRPS28 acts as a competing endogenous RNA, sponging chi-miR-145-5p, thereby relieving its inhibitory effect on the target gene *NUAK1*. This, in turn, inhibits the formation of dermal papilla structures and ultimately affects the morphogenesis of secondary hair follicles during the embryonic period. This study provides a novel perspective on dissecting the regulatory network of hair follicle development in cashmere goats and offers potential theoretical insights for breeding new strains of cashmere goats.

## Simple Summary

The development of secondary hair follicles in cashmere goats directly influences the yield and quality of cashmere, and hair follicle morphogenesis is a complex biological process involving multiple signaling pathways and regulatory factors. Through specific screening and analysis, this study identified lncRNA MRPS28, which exhibits significant differential expression during the morphogenesis of secondary hair follicles. The results indicate that lncRNA MRPS28 can inhibit the proliferation and migration of dermal fibroblasts via the chi-miR-145-5p/NUAK1 axis, thereby impeding the formation of dermal papilla structure and ultimately affecting the morphogenesis of secondary hair follicles during the embryonic stage. This study is the first to elucidate the molecular mechanism by which lncRNA MRPS28 regulates the development of secondary hair follicles in cashmere goats via the chi-miR-145-5p/NUAK1 axis. It offers a novel perspective on dissecting the regulatory network of hair follicle development in cashmere goats and provides potential theoretical insights into breeding new strains of cashmere goats.

## 1. Introduction

Cashmere goats are an important economic livestock species renowned for producing high-quality cashmere. They are widely distributed globally and have a long history of breeding, particularly in countries such as China, Mongolia, and Iran [1]. They have a medium build and strong adaptability, enabling them to survive and reproduce in harsh environmental conditions [1]. The cashmere produced by cashmere goats has a soft texture and excellent warmth retention [2]. It is praised as the “soft gold” or “fiber gemstone” and serves as a precious raw material for high-end textiles [2]. Textiles made from cashmere goat hair, including cashmere sweaters, scarves, and coats, are favored by consumers for their lightweight, warm, and comfortable wearing experience, and enjoy a great reputation in the international market [3]. In recent years, with the increasing demand for high-quality natural fibers, the market demand for cashmere has continued to rise, driving the rapid development of the cashmere goat industry [3]. However, the production and quality of cashmere goat hair are influenced by various factors, with the morphological development of secondary hair follicles (SHFs) during the embryonic period being crucial in determining cashmere yield [3]. Therefore, in-depth research into the regulatory mechanisms of SHF development in cashmere goats is of great significance for improving cashmere yield and quality and promoting the sustainable development of the cashmere goat industry.

Hair follicles are crucial appendages of the skin, located within the dermis, and serve as key organs regulating hair growth, shedding, and regeneration [1]. SHFs produce cashmere, and their degree of development directly determines the yield and quality of cashmere [2]. The development of cashmere goat hair follicles begins in the embryonic period and is a highly complex and orderly process [3]. Studies have shown that the development of primary hair follicles (PHFs) in cashmere goats precedes that of SHFs [3]. Specifically, by embryonic day 45, the fetal skin has formed a complete epidermal structure; by day 55, PHFs begin to develop, with hair bud structures emerging; by day 65, PHF hair buds continue to grow and penetrate deeper into the dermal layer of the skin; and by day 75, SHFs begin to develop, with SHF hair bud structures appearing for the first time [3,4]. Notably, the developmental state of SHFs during the embryonic period directly determines the cashmere density and yield of cashmere goats after birth [4]. Therefore, in-depth research into the developmental process of SHFs in cashmere goats during the embryonic period is of great significance for revealing the molecular mechanisms of cashmere formation, screening for key regulatory factors, and improving the yield and quality of cashmere.

Hair follicle development relies on precise signaling between the dermis and epidermis. The dermal papilla (DP), which serves as the signaling hub of hair follicles, is derived from the proliferation and differentiation of dermal fibroblasts and is a crucial structure for ensuring normal hair follicle growth [2]. Studies have shown that embryonic hair follicle development is precisely regulated by various signaling pathways and regulatory factors, including Wnt, BMP, and FGF signaling pathways, as well as miRNAs and lncRNAs [5]. LncRNAs, which were once considered “noise” in the transcription process, have recently been found to play important regulatory roles in hair follicle development and hair growth [5]. For example, lncRNA MSTRG.20890.1 and lncRNA MSTRG.14227.1 can inhibit the expression of the *ADAMTS3* gene by targeting miRNAs, thereby inhibiting the proliferation and migration of dermal fibroblasts in cashmere goats [1,2]. LncRMA-627.1 can competitively bind to miR-29a-5p, which is also targeted by *EDAR*, thereby regulating the formation of the hair follicle placode [6]. LncRNA FABP_AS promotes *DKK1* gene expression by competitively binding to chi-miR-335-5p, inhibiting the activity of the Wnt/β-catenin signaling pathway, and thereby regulating the proliferation of hair follicle stem cells [7]. In addition, Zhang et al. identified 521 differentially expressed lncRNAs in the embryonic skin of cashmere goats through high-throughput sequencing technology, with lncRNA H19, in particular, significantly promoting the viability and proliferation of DP cells through the chi-miR-214-3p/β-catenin axis [8]. Although studies have gradually revealed some key signaling molecules regulating hair follicle development in cashmere goats, the specific mechanisms of lncRNAs in SHF development remain poorly understood and require further exploration.

Based on the embryonic skin transcriptome database of cashmere goats previously constructed by our research group [3], this study screened 158 lncRNAs related to SHF morphogenesis. Further analysis revealed that lncRNA MRPS28, transcribed from the intron region of the *MRPS28* gene, is significantly underexpressed at the critical stage of SHF development (embryonic day 75). Functional studies showed that lncRNA MRPS28 can sponge chi-miR-145-5p, relieving its inhibitory effect on the *NUAK1* gene, thereby upregulating *NUAK1* expression and inhibiting the proliferation and migration of dermal fibroblasts. However, this inhibitory effect may be achieved by reducing the proportion of cells in the S-phase of the cell cycle. This study aims to deeply explore the molecular mechanism by which lncRNA MRPS28 regulates the proliferation and migration of dermal fibroblasts through the chi-miR-145-5p/NUAK1 axis, and thereby affects the morphogenesis of SHFs in cashmere goats, so as to enrich the regulatory network of lncRNA in the development of SHFs in cashmere goats and provide a theoretical basis and molecular targets for improving the quality and yield of cashmere from cashmere goats.

## 2. Materials and Methods

### 2.1. Skin Sample Collection

The experimental animals utilized in the study were obtained from the Inner Mongolia Jinlai Animal Husbandry Technology Co., Ltd. (Hohhot, China). Twelve three-year-old ewes of Inner Mongolia cashmere goats with good production performance and the same growth environment were selected for synchronous estrus treatment. According to the insemination records of the ewes, skin tissue samples were collected from the fetuses of cashmere goats at different developmental stages (45, 55, 65, and 75 days), with three fetuses sampled at each stage. To ensure the preservation of their biological integrity and to maintain the quality of the samples for future analysis, these skin samples were promptly stored at −80 °C.

### 2.2. Screening of LncRNAs Related to Secondary Hair Follicle Morphogenesis

We utilized the established transcriptome database of cashmere goat skin from different embryonic stages (45, 55, 65, and 75 days) to identify lncRNAs that were differentially expressed with a |log2foldchange| ≥ 1 and *p* ≤ 0.05 [3]. Based on the characteristics of SHFs’ morphogenesis [4], we designated d55vsd45, d65vsd45, and d65vsd55 as Stage 1, which is related to the development of PHFs. Similarly, we designated d75vsd45, d75vsd55, and d75vsd65 as Stage 2, all of which are associated with the development of both PHFs and SHFs. Subsequently, we identified the intersection of Stage 1 and Stage 2 and excluded this intersection from the definition of Stage 2, leaving the remaining part of Stage 2 as the important lncRNAs specifically related to the development of SHFs.

### 2.3. Subcellular Localization

LncLocator 1.0 software [9] was used to predict the subcellular location of the lncRNA MRPS28. Subsequently, the cytoplasm and nucleus of the dermal fibroblasts were separated using a cytoplasmic and nuclear RNA purification kit (Norgen Biotek, Thorold, ON, Canada), and RNA was extracted from both. Following extraction, qRT-PCR was performed to detect the expression levels of lncRNA MRPS28 in the cytoplasmic and nuclear fractions.

### 2.4. FISH Assay

The dermal fibroblasts were adjusted to the desired density. Subsequently, the cells were fixed with 4% paraformaldehyde for 20 min, treated with Hyb buffer reagent for 1 h, and incubated overnight with the mixture of Hyb buffer and the probe. Afterward, the prehybridization solution was removed, and blocking serum was added. The cells were then incubated with BSA for 30 min at room temperature. Lastly, the cells were stained with DAPI solution for 8 min, washed to remove the staining solution, and examined under a fluorescence microscope.

### 2.5. Dual-Luciferase Report Detection

The dual-luciferase reporting system was used to verify the targeting relationship between a miRNA and its target gene. Specifically, miRNA mimics were co-transfected with psiCHECK2 vectors containing either wild-type (WT) or mutant (MUT) sequences of the target gene, using LipoFiter as the transfection reagent (Hanheng, Shanghai, China). Luciferase activity was measured 48 h post-transfection to assess the interaction between the miRNA and its target sequence. In the dual-luciferase assay, the wild-type plasmid refers to the one containing the original sequence of the target gene, which is used to simulate the normal function of the gene. The mutant plasmids are artificially modified sequences of the target gene (such as point mutations or deletions), which are employed to investigate the impact of sequence alterations on gene function.

### 2.6. Cell Culture and Transfection

Dermal fibroblasts were cultured in a medium containing 10% fetal bovine serum, essential for their growth. To ensure optimal conditions for cell growth and proliferation, all cell lines were maintained at 37 °C with 5% CO_2_. Based on the sequence information of lncRNA MRPS28 and *NUAK1*, the interference vectors targeting lncRNA MRPS28 and *NUAK1* were constructed, respectively (Hanheng, Shanghai, China). Then, these interference vectors were transfected into dermal fibroblasts using lentivirus-mediated transfection. After transfection, the successfully transfected dermal fibroblasts were screened with purinomycin. During the experiment, we regarded the dermal fibroblasts that were not transfected as the blank control (NC). Meanwhile, we considered the dermal fibroblasts transfected with the empty vector as the negative control (sh-NC), and those transfected with the target interference sequence as the experimental group.

### 2.7. Cell Proliferation Assays

The proliferation of cells was evaluated through two methods: CCK8 and EdU. The CCK8 kit (Solarbio, Beijing, China) was utilized to assess cell proliferation, with the absorbance value of each well determined using an enzyme-labeling instrument at a wavelength of 450 nm. Additionally, the EdU assay was performed according to the guidelines provided by the BeyoClick™ EdU-555 Cell Proliferation Detection Kit (Beyotime, Shanghai, China). This assay determined the proliferation rate by calculating the ratio of cells labeled with EdU to those labeled with Hoechst.

### 2.8. DNA Staining

The cell cycle was determined using the DNA content quantification method (Solarbio, Beijing, China). To prepare the cells for analysis, they were first fixed in 70% ethanol, resuspended, and incubated at 4 °C. After a 24-hour incubation, RNase A was added, and the cells were further incubated at 37 °C for 30 min. Subsequently, a propidium iodide staining solution was added to stain the DNA. The cells were then incubated at 4 °C in the dark for an additional 30 min to ensure adequate staining, and the cell cycle was detected by flow cytometry (CytoFLEX, Beckman, Brea, CA, USA).

### 2.9. Cell Apoptosis Detection

Cell apoptosis was determined using the Annexin V-APC/PI Kit (Elabscience Biotechnology, Wuhan, China). A single-cell suspension was prepared and stained with reagents. After incubating in the dark for 20 min, cell apoptosis was detected by flow cytometry (CytoFLEX, Beckman, Brea, CA, USA).

### 2.10. Cell Scratch Assay

The cell scratch assay was conducted by scraping a monolayer of cells to create a wound [1]. Images of the wounded area were captured immediately after scraping (0 h post-scratch) and again after a 24-hour incubation period. Before scraping, the cells were washed with PBS to remove any debris or unattached cells. Following the scratch, fresh serum-free medium was added to the cells, which were then incubated under standard conditions for 24 h. Cell migration into the wounded area was evaluated by comparing the images taken at 0 h and 24 h post-scratch. The cell migration rate was calculated using the formula: [(0 h area − 24 h area)/0 h area] × 100%.

### 2.11. Statistical Analysis

Statistical analysis was conducted using SPSS 26.0 (IBM, SPSS, Chicago, IL, USA) and GraphPad Prism 8.0 (GraphPad Software Inc., San Diego, CA, USA). All data are presented as mean ± SD from ≥3 independent experiments. Student’s *t*-tests determined significance with *p* < 0.05 or *p* < 0.01.

## 3. Results

### 3.1. Identifying LncRNAs Associated with the Development of Secondary Hair Follicles in Cashmere Goats

Based on the previously constructed transcriptome database of skin tissues from different embryonic stages (45, 55, 65, and 75 days) of cashmere goats [3], we conducted an in-depth analysis of lncRNA expression across these embryonic stages and successfully identified 1209 differentially expressed lncRNAs. The skin of cashmere goats contains two types of hair follicles: primary and secondary, both of which initiate development during the embryonic period and complete their maturation after birth. Notably, previous studies have shown that the development of PHFs precedes that of SHFs, with PHFs occurring at embryonic day 55 and SHFs at embryonic day 75, respectively [4].

**Figure 1:**
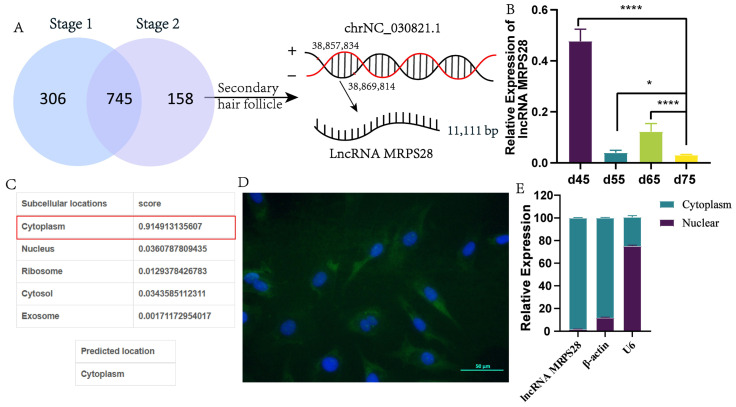
Screening and identification of lncRNA related to SHF morphogenesis. (**A**) Screening of lncRNAs related to SHF morphogenesis. (**B**) Expression of lncRNA MRPS28 in skin tissues at different embryonic periods. (**C**) The lncLocator software 1.0 predicts the distribution of lncRNA MRPS28 in cells. (**D**) The distribution of lncRNA MRPS28 in cells was detected by FISH assay. (**E**) Detection of lncRNA MRPS28 expression in the nucleus and cytoplasm of dermal fibroblasts. Note: * *p* < 0.05, and **** *p* < 0.0001 indicate the levels of significance. ns indicates that the difference is not significant.

Considering the developmental characteristics of PHFs and SHFs in cashmere goats, we classified the three comparison groups (d55 vs d45, d65 vs d45, and d65 vs d55) as Stage 1, and in these groups, we found a total of 1051 lncRNAs related to the development of PHFs. Simultaneously, the three comparison groups (d75 vs d45, d75 vs d55, and d75 vs d65) were classified as Stage 2, and in these groups, we found a total of 903 lncRNAs related to the development of both PHFs and SHFs. By taking the intersection of Stage 1 and Stage 2, and then excluding this intersection from Stage 2, we identified 158 key lncRNAs that are likely closely related to the development of SHFs (Figure 1A).

Among these 158 lncRNAs, we focused specifically on lncRNA MRPS28, which has a full length of 11,111 bp and is situated within the intron region of the *MRPS28* gene. Notably, lncRNA MRPS28 showed significantly low expression levels during the critical period of SHF morphogenesis (embryonic day 75) (Figure 1A,B). Therefore, we hypothesize that lncRNA MRPS28 may play a role in the morphogenesis of SHFs in cashmere goats.

Additionally, we analyzed the coding capacity and cellular localization of lncRNA-MRPS28. Bioinformatics predictions revealed CPC and CNCI scores of 0.13 and −0.07 for lncRNA-MRPS28, respectively, confirming its identity as a non-coding lncRNA. Meanwhile, lncLocator predictions suggested that this lncRNA is primarily localized in the cytoplasm (Figure 1C). To confirm this prediction, we conducted FISH experiments and nuclear–cytoplasmic separation assays. Both experimental results demonstrated that the expression level of lncRNA-MRPS28 in the cytoplasm was much higher than that in the nucleus, which may be closely related to its primary regulatory function (Figure 1D,E).

### 3.2. The Role of LncRNA MRPS28 in Inhibiting the Proliferation and Migration of Dermal Fibroblasts

**Figure 2:**
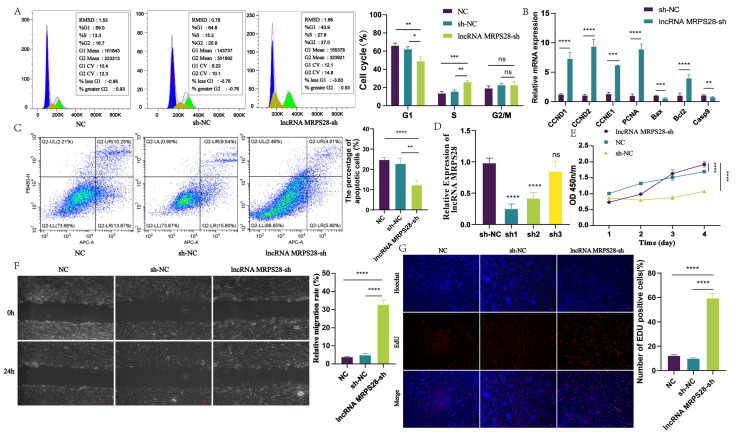
Functional analysis of lncRNA MRPS28 in dermal fibroblasts. (**A**) DNA staining was used to detect the cell cycle of lncRNA MRPS28−sh cell line. (**B**) qRT−PCR detected the expression of cell proliferation/apoptosis marker genes. (**C**) The apoptosis of dermal fibroblasts was detected after lncRNA MRPS28 interference. (**D**) Screening of lncRNA MRPS28 interference vector. (**E**) CCK8 was used to detect the proliferation of lncRNA MRPS28−sh cell line. (**F**) The cell scratch assay was used to detect lncRNA MRPS28−sh cell line migration. (**G**) EdU was used to detect the proliferation of lncRNA MRPS28−sh cell line. Note: * *p* < 0.05, ** *p* < 0.01, *** *p* < 0.001, and *** *p* < 0.0001 indicate the levels of significance. ns indicates that the difference is not significant.

There exists a close relationship between dermal fibroblasts and DP cells. During embryogenesis, dermal fibroblasts undergo continuous proliferation and differentiation, ultimately forming the DP structure [2]. This structure serves as the signaling hub of hair follicles, playing a pivotal role in guiding and regulating hair follicle development and cyclic growth [1]. In this study, we constructed three lncRNA MRPS28 interference vectors (sh1, sh2, sh3) and transfected them into dermal fibroblasts using lentiviral transfection. Subsequently, we extracted cellular RNA and examined the expression level of lncRNA MRPS28. The results showed that both sh1 and sh2 significantly inhibited the expression of lncRNA MRPS28 in cells, with sh1 exhibiting the best interference efficiency (Figure 2D). Therefore, we selected lncRNA MRPS28-sh1 for subsequent experiments.

During hair follicle morphogenesis, dermal fibroblasts undergo continuous proliferation to facilitate the formation of the subsequent DP structure. In this study, we investigated the effect of lncRNA MRPS28 on the proliferation of dermal fibroblasts using CCK8 and EdU assays. The proliferation results revealed that following lncRNA MRPS28 interference, the proportion of EdU-positive cells significantly increased, indicating enhanced cellular proliferation (Figure 2E,G). Annexin V-APC/PI double staining experiments demonstrated that the apoptotic rate of cells in the lncRNA MRPS28-sh cell line was significantly lower compared to the control group (Figure 2C). To further explore the proliferative effect of lncRNA MRPS28-sh on cells, we examined changes in the cell cycle and the expression of genes related to cell proliferation and apoptosis. The experimental results showed that interference with lncRNA MRPS28 increased the proportion of S-phase cells in dermal fibroblasts (Figure 2A), promoted the expression of proliferation-related marker genes such as *CCND1*, *CCND2*, and *PCNA*, and inhibited the expression of apoptosis-related genes such as *Bax* and *Casp9* (Figure 2B). These results indicate that interfering with lncRNA MRPS28 enhances the proliferation of dermal fibroblasts, potentially by increasing the proportion of S-phase cells and upregulating the expression of proliferation-related genes.

During hair follicle development, dermal fibroblasts not only proliferate but also migrate deeper into the dermis, eventually forming the DP structure at the base of the hair follicle. Consequently, cellular migration ability is a crucial indicator when exploring the formation of DP by dermal fibroblasts. In this study, we further assessed cell migration by measuring the scratch area of different cell lines at 0 h and 24 h. The experimental results revealed that, under the same serum-free culture conditions, the scratch area of the lncRNA MRPS28-sh cell line was significantly smaller than that of the control group, indicating that interference with lncRNA MRPS28 enhances the migration of dermal fibroblasts (Figure 2F).

In summary, the above experimental results indicate that interference with lncRNA MRPS28 promotes the proliferation and migration of dermal fibroblasts, thereby influencing the morphogenesis of SHFs. However, its proliferative effect on dermal fibroblasts may be mediated by increasing the proportion of S-phase cells and promoting the expression of proliferation-related marker genes.

### 3.3. Constructing the Regulatory Network of LncRNA MRPS28/chi-miR-145-5p/NUAK1

Studies have indicated that the subcellular localization of lncRNAs within cells is associated with their primary regulatory functions [5,10]. Typically, lncRNAs located in the cytoplasm often indirectly regulate the expression of related genes by acting as miRNA sponges. Preliminary experimental results indicate that lncRNA MRPS28 is primarily located in the cytoplasm of cells. Based on this, we hypothesize that lncRNA MRPS28 may function as a ceRNA to regulate the expression of related genes.

**Figure 3:**
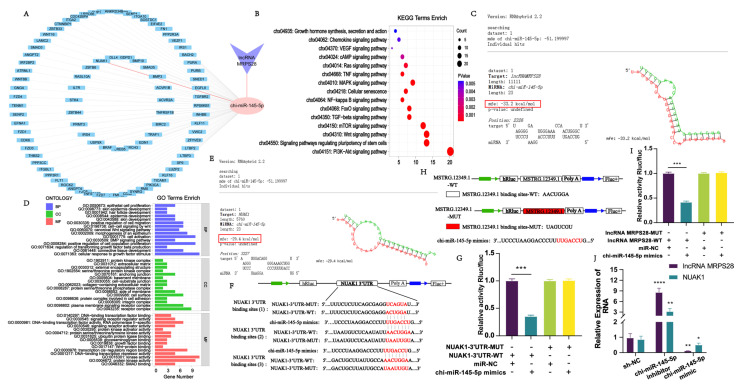
Construction and verification of lncRNA MRPS28/chi-miR-145-5p/NUAK1 regulatory network. (**A**) Construction of a regulatory network. (**B**) KEGG enrichment analysis of chi-miR-145-5p target genes. (**C**) RNAhybrid (v2.1.2) software predicts the sequence of lncRNA MRPS28 binding site to chi-miR-145-5p. (**D**) GO enrichment analysis of chi-miR-145-5p target genes. (**E**) RNAhybrid (v2.1.2) software predicts the sequence of *NUAK1* binding site to chi-miR-145-5p. (**F**) Schematic diagram of wild-type/mutant-type NUAK1-3’UTR luciferase reporter vector construction. (**G**) Dual-luciferase reporter gene system to detect target binding of chi-miR-145-5p to NUAK1-3’UTR. (**H**) Schematic diagram of wild/mutant lncRNA MRPS28 luciferase reporter vector construction. (**I**) Dual-luciferase reporter gene system to detect target binding of lncRNA MRPS28 to chi-miR-145-5p. (**J**) chi-miR-145-5p interference/overexpression of dermal fibroblast cell lines to detect lncRNA MRPS28/NUAK1 expression. Note: * *p* < 0.05, ** *p* < 0.01, *** *p* < 0.001, and **** *p* < 0.0001 indicate the levels of significance. ns indicates that the difference is not significant.

To validate this hypothesis, we initially performed bioinformatics analyses using TargetScan and miRanda and found potential binding sites between lncRNA MRPS28 and chi-miR-145-5p. Subsequently, we used RNAhybrid (v2.1.2) software to obtain sequence information about the binding sites between lncRNA MRPS28 and chi-miR-145-5p. The results showed that the binding free energy between them was −33.2 kcal/mol, which is lower than the threshold of −20 kcal/mol, indicating a strong binding affinity between them (Figure 3C). To further confirm the targeting relationship, we constructed both wild-type and mutant dual-luciferase reporter plasmids of lncRNA MRPS28 and utilized the dual-luciferase reporter gene system for detection (Figure 3H). The results demonstrated that lncRNA MRPS28 is capable of binding to chi-miR-145-5p (Figure 3I).

Meanwhile, we predicted the target genes of chi-miR-145-5p and identified potential binding sites with 88 mRNAs, and the regulatory network is shown in Figure 3A. Next, we performed an enrichment analysis on these target genes. The results of the enrichment analysis revealed that these genes were primarily enriched in signaling pathways associated with hair follicle development, including Wnt, TGF-β, PI3K-Akt, and FoxO (Figure 3B,D). The FoxO signaling pathway plays a crucial role in organisms and is extensively involved in multiple important physiological processes, such as cell cycle regulation, apoptosis, and metabolic regulation [11]. Studies have demonstrated that several members of the Fox family, including *FOXM1*, *FOXN1*, *FOXE1*, and *FOXI3*, are all related to hair follicle growth, development, and hair formation [12,13,14,15]. Through our analysis of the 88 target genes, we discovered that the *NUAK1* gene was enriched in the FoxO signaling pathway and plays a significant role in this pathway. Therefore, we selected this gene for further research. Both RNAhybrid and the dual-luciferase reporter gene system indicated the existence of binding sites for chi-miR-145-5p within the 3’UTR region of *NUAK1* (Figure 3E–G).

Subsequently, we employed qRT-PCR to assess the expression levels of lncRNA MRPS28 and *NUAK1* in cell lines with either chi-miR-145-5p knockdown or overexpression. The results revealed that both lncRNA MRPS28 and *NUAK1* exhibited higher expression in the chi-miR-145-5p knockdown cell line, whereas they showed lower expression in the chi-miR-145-5p overexpression cell line (Figure 3J). This phenomenon is consistent with the ceRNA hypothesis. In summary, we have further investigated the regulatory mechanism of lncRNA MRPS28 and found that, in dermal fibroblasts, lncRNA MRPS28 can competitively bind to chi-miR-145-5p with *NUAK1*, thus modulating the expression of *NUAK1*.

### 3.4. Inhibitory Role of NUAK1 on the Proliferation and Migration of Dermal Fibroblasts

**Figure 4:**
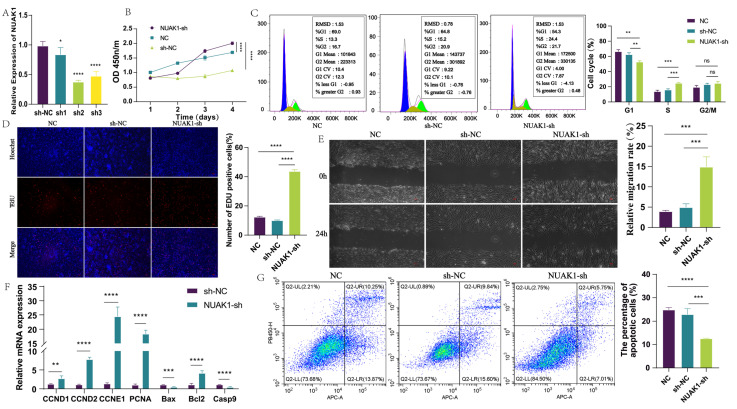
Functional analysis of *NUAK1* in dermal fibroblasts. (**A**) Screening of *NUAK1* interference vector. (**B**) CCK8 was used to detect the proliferation of NUAK1-sh cell line. (**C**) DNA staining was used to detect the cell cycle of lncRNA NUAK1-sh cell line. (**D**) EdU was used to detect the proliferation of NUAK1-sh cell line. (**E**) Cell scratch assay was used to detect the migration of NUAK1-sh cell line. (**F**) qRT-PCR detected the expression of cell proliferation/apoptosis marker genes. (**G**) The apoptosis of dermal fibroblasts was detected after *NUAK1* interference. Note: * *p* < 0.05, ** *p* < 0.01, *** *p* < 0.001, and **** *p* < 0.0001 indicate the levels of significance. ns indicates that the difference is not significant.

In addition, based on the sequence information of *NUAK1*, three *NUAK1* interference vectors (sh1, sh2, sh3) were constructed, and the three vectors were transfected into dermal fibroblasts using lentivirus-mediated transfection. After testing, NUAK1-sh2 exhibited the best knockdown efficiency, thus we selected it for subsequent experiments (Figure 4A). After completing the foundational work of cell line screening, we turned our focus to cell proliferation, a critical cellular activity, and conducted cell proliferation experiments first. The results of the cell proliferation experiments demonstrated that, following *NUAK1* knockdown, the proportion of EdU-positive cells in dermal fibroblasts significantly increased, indicating a marked enhancement in cellular proliferation capacity (Figure 4B,D). To further validate this finding, we conducted cell apoptosis assays. Consistent with the proliferation results, the apoptosis experiments showed that after *NUAK1* knockdown, the apoptotic rate of dermal fibroblasts significantly decreased (Figure 4G). This suggests that *NUAK1* knockdown significantly enhances the proliferation capacity of dermal fibroblasts and inhibits their apoptosis.

The basic life activities of dermal fibroblasts, including proliferation and apoptosis, do not occur in isolation. They are closely intertwined with the cell cycle and migration ability, collectively determining cellular behavior and fate. In light of this, we further explored the effects of *NUAK1* on cell cycle distribution and cell migration capacity. The results of cell scratch experiments showed that the migration capacity of the experimental group with NUAK1-sh was significantly enhanced compared to that of the control group (Figure 4E). This finding demonstrates that *NUAK1* knockdown effectively promotes the migration of dermal fibroblasts. Moreover, the results of cell cycle experiments demonstrated that following *NUAK1* knockdown, the proportion of dermal fibroblasts in the S-phase significantly increased. This suggests that the proliferative promotion effect of NUAK1-sh may be mediated by increasing the proportion of cells in the S-phase, thereby accelerating cell mitosis (Figure 4C). Additionally, we employed qRT-PCR to assess changes in the expression of markers associated with cell proliferation and apoptosis. The results indicated that NUAK1-sh promoted the expression of markers related to cell proliferation and anti-apoptosis, while simultaneously inhibiting the expression of pro-apoptotic genes (Figure 4F).

Based on the above experimental results, which include cell line construction and screening, exploration of various cellular behaviors such as cell proliferation, apoptosis, migration, and cell cycle, as well as analysis at the gene expression level, we can conclude that *NUAK1* knockdown promotes the proliferation and migration capacity of dermal fibroblasts. This promotional effect is attained by accelerating the mitosis of dermal fibroblasts. Notably, upon reviewing our previous research on lncRNA MRPS28-sh, we observed a high degree of consistency between the effects of NUAK1-sh and those of lncRNA MRPS28-sh on the phenotypes of dermal fibroblasts. This phenomenon is in good alignment with the ceRNA hypothesis, thus providing strong evidence for our in-depth understanding of the complex intracellular regulatory networks.

### 3.5. Chi-miR-145-5p Inhibitor Reverses the Inhibitory Effects on Dermal Fibroblast Proliferation and Migration That Are Mediated by LncRNA MRPS28 and NUAK1

To further validate the lncRNA MRPS28/chi-miR-145-5p/NUAK1 regulatory network, we conducted a series of rescue experiments. Previously, we had already elucidated the effects of lncRNA MRPS28-sh and NUAK1-sh on dermal fibroblasts through numerous experiments. The previous experimental results clearly showed that both lncRNA MRPS28-sh and NUAK1-sh could promote the proliferation and migration of dermal fibroblasts, while simultaneously inhibiting their apoptosis. Based on these findings, we decided to shift our research focus to the key component of this regulatory network: chi-miR-145-5p.

**Figure 5:**
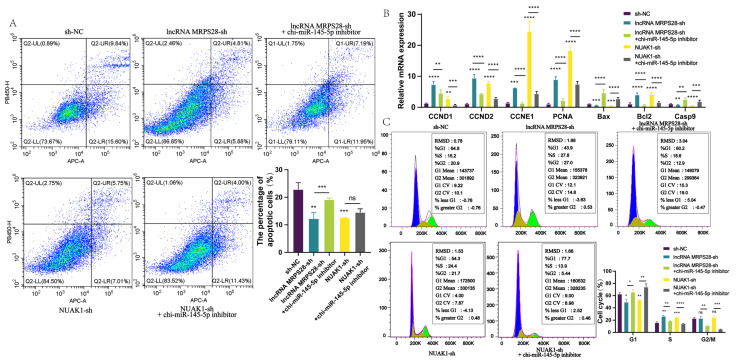
Chi-miR-145-5p can reverse the effect of lncRNA MRPS28-sh/NUAK1-sh on the cell phenotype of dermal fibroblasts. (**A**) Annexin V-APC/PI double staining was used to detect cell apoptosis. (**B**) qRT-PCR detected the expression of cell proliferation/apoptosis marker genes. (**C**) DNA staining was used to detect the cell cycle. Note: * *p* < 0.05, ** *p* < 0.01, *** *p* < 0.001, and **** *p* < 0.0001 indicate the levels of significance. ns indicates that the difference is not significant.

**Figure 6:**
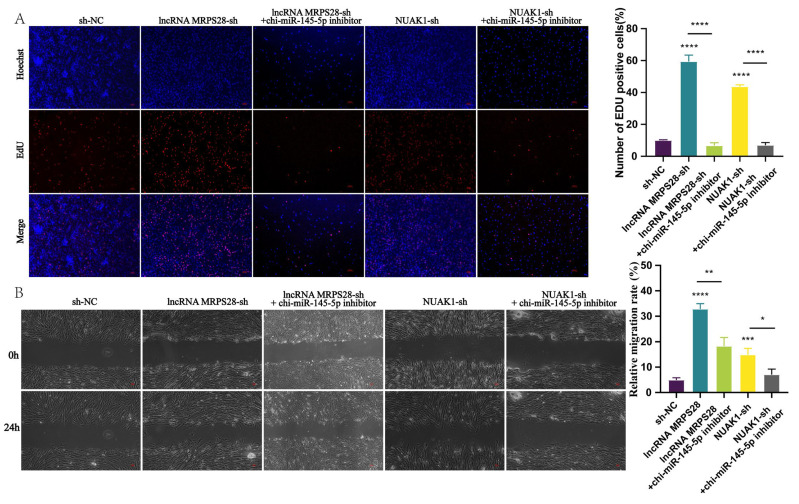
Chi-miR-145-5p can reverse the effect of lncRNA MRPS28-sh/NUAK1-sh on the cell phenotype of dermal fibroblasts. (**A**) EdU assay was used to detect cell proliferation. (**B**) Cell scratch assay was used to detect cell migration. Note: * *p* < 0.05, ** *p* < 0.01, *** *p* < 0.001, and **** *p* < 0.0001 indicate the levels of significance. ns indicates that the difference is not significant.

In this experiment, we added chi-miR-145-5p inhibitors to both the lncRNA MRPS28-sh and NUAK1-sh cell lines, aiming to observe changes in cell phenotypes and thereby delve deeper into the underlying mechanisms of this regulatory network. After successfully adding chi-miR-145-5p inhibitors to the lncRNA MRPS28-sh and NUAK1-sh cell lines, we observed a significant decrease in the proportion of EdU-positive cells in the co-transfected cell lines, accompanied by a marked reduction in the expression of genes related to cell proliferation (Figure 5B and Figure 6A). Meanwhile, cell cycle results indicated that the proportion of cells in the S-phase also decreased significantly in the co-transfected cell lines (Figure 5C). This series of data strongly suggests that chi-miR-145-5p inhibitors can effectively counteract the proliferative effect on dermal fibroblasts promoted by lncRNA MRPS28-sh and NUAK1-sh. After clarifying the impact on cell proliferation, we promptly investigated cell apoptosis and found that the addition of chi-miR-145-5p inhibitors significantly increased the proportion of apoptotic cells in both the lncRNA MRPS28-sh and NUAK1-sh cell lines, accompanied by a notable upregulation of pro-apoptotic genes (Figure 5A,B). This result further indicates that chi-miR-145-5p inhibitors can not only counteract the proliferative effect of dermal fibroblasts promoted by lncRNA MRPS28-sh and NUAK1-sh but also reverse their suppressive effect on cell apoptosis. In terms of cell migration, Figure 6B enables us to intuitively observe that the promotive effect on cell migration conferred by lncRNA MRPS28-sh and NUAK1-sh was gradually counteracted upon the addition of chi-miR-145-5p inhibitors to these cell lines.

**Figure 7:**
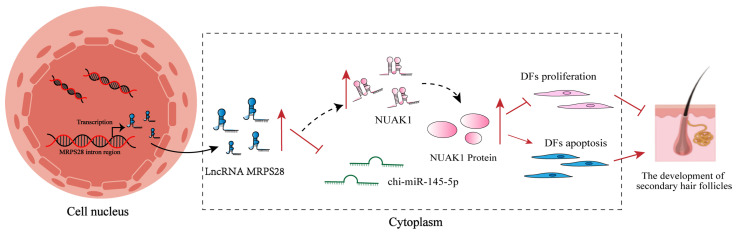
Diagram of the lncRNA MRPS28/chi-miR-145-5p/NUAK1 regulatory mechanism.

Based on the above results, we can confidently conclude that chi-miR-145-5p, as a common target miRNA for both lncRNA MRPS28 and *NUAK1*, can have its inhibitors rescue the promotional effects on cell proliferation and migration induced by lncRNA MRPS28-sh and NUAK1-sh. Additionally, in dermal fibroblasts, lncRNA MRPS28 acts as a sponge for chi-miR-145-5p, regulating the expression of the downstream target gene *NUAK1* to inhibit the proliferation and migration of dermal fibroblasts, thereby playing a crucial role in the morphogenesis of SHFs (Figure 7).

## 4. Discussion

Cashmere goats are an important economic animal, and the quality and yield of their cashmere are directly related to the financial benefits of the breeding industry [3]. The growth and development of cashmere is a complex biological process that involves the regulation of multiple genes and signaling pathways [2]. In recent years, thanks to the rapid development of molecular biology techniques, the role of lncRNA in hair follicle development has increasingly become a research focus [5]. Studies have demonstrated that lncRNA-HOTAIR is expressed throughout both the growth and resting phases of SHFs in cashmere goats, with expression levels significantly higher during the growth phase compared to the resting phase [16]. This suggests its potential involvement in the reconstruction of SHFs and the formation and growth of cashmere [16]. Additionally, lncRNA-H19 significantly promotes the viability and proliferation of DP cells, and this regulatory effect is mediated through the chi-miR-214-3p/β-catenin axis [8]. Furthermore, lncRNA PlncRNA-1 may enhance the proliferation and differentiation of hair follicle stem cells by upregulating the Wnt/β-catenin signaling pathway mediated by *TGF-β1* [11].

In this study, we screened 158 lncRNAs related to SHF morphogenesis from a transcriptome database of skin samples collected from cashmere goats at different embryonic stages, which we had previously constructed [3]. Among these, lncRNA MRPS28, transcribed from the intron region of the *MRPS28* gene, exhibited significantly low expression during SHF morphogenesis. Through experimental analysis and validation, we discovered that lncRNA MRPS28 can act as a sponge for chi-miR-145-5p, effectively alleviating the inhibitory effect of chi-miR-145-5p on *NUAK1* expression in dermal fibroblasts.

Hair follicles, which are important appendages of mammalian skin, are composed of various cell types and play a crucial role in hair growth, replacement, and the maintenance of skin’s physiological functions [2]. During hair follicle development, dermal fibroblasts are the most crucial participants, ultimately differentiating into the DP, a structure that serves as the “signaling hub” of hair follicles [2]. This structure directly regulates hair follicle development, cyclic growth, and hair production through intercellular interactions and signal transmission [2]. In light of this, we investigated the effects of lncRNA MRPS28 on the phenotypes of dermal fibroblasts and found that it is primarily located in the cytoplasm. Interference with lncRNA MRPS28 significantly enhanced the proliferation and migration capabilities of dermal fibroblasts. In recent years, an increasing number of studies have shown that lncRNAs play extensive regulatory roles in organisms, one of which is their important function as sponge molecules that regulate miRNAs. For example, lncRNA MSTRG.20890.1 upregulates *ADAMTS3* expression by competitively binding to chi-miR-24-3p, thereby promoting the proliferation of dermal fibroblasts [1]; lncRNA-XIST competes with *Shh* for binding to miR-424, activating the Hedgehog signaling pathway and promoting dermal papilla-mediated hair follicle regeneration [12]. Additionally, lncRNA FABP_AS competitively binds to chi-miR-335-5p, which promotes *DKK1* gene expression and reduces Wnt/β-catenin signaling pathway activity, thereby inhibiting the proliferation of hair follicle stem cells [7]. These studies all indicate that lncRNAs can indirectly regulate hair follicle development by interacting with various key factors and signaling pathways.

In this study, we employed bioinformatics methods to predict the miRNAs that are targeted by lncRNA MRPS28, as well as the genes targeted by these miRNAs. Through analysis, we found that lncRNA MRPS28 has a targeted binding site for chi-miR-145-5p. chi-miR-145-5p is one of the important miRNAs during the growth and development of hair follicles. Previous studies have demonstrated that chi-miR-145-5p can promote the differentiation of hair follicle stem cells by regulating the expression of the *NANOG* and *Sox9* genes [13]. Given these findings, in this study, we targeted chi-miR-145-5p as the miRNA of interest to further predict its target genes. Through bioinformatics analysis, it was revealed that chi-miR-145-5p has potential binding sites in 88 genes. Enrichment analysis of the target genes of chi-miR-145-5p found that they are mainly enriched in Wnt, TGF-β, PI3K-Akt, and FoxO signaling pathways, which are related to hair follicles. The FoxO signaling pathway is crucial in organisms, widely participating in multiple important physiological processes such as cell cycle regulation, cell apoptosis, and metabolic regulation [14]. Studies have shown that among the Fox family, *FOXM1*, *FOXN1*, *FOXE1*, and *FOXI3* are associated with hair follicle growth, development, and hair formation [15,17,18,19]. Among the 88 target genes, we found that the *NUAK1* gene is enriched in the FoxO signaling pathway and plays an important role in this pathway. Therefore, we selected this gene for further research. The results of qRT-PCR and dual-luciferase reporter assays showed that both lncRNA MRPS28 and *NUAK1* have binding sites for chi-miR-145-5p, indicating the existence of the lncRNA MRPS28/chi-miR-145-5p/NUAK1 regulatory network. Furthermore, we validated this regulatory network at the cellular level. By adding chi-miR-145-5p inhibitors to both the lncRNA MRPS28-sh and NUAK1-sh cell lines, we found that the promotional effects on dermal fibroblast proliferation and migration, as well as the inhibitory effect on apoptosis, caused by lncRNA MRPS28-sh and NUAK1-sh, could be counteracted by the chi-miR-145-5p inhibitor. This further demonstrates that in cashmere goat dermal fibroblasts, lncRNA MRPS28 indeed inhibits the proliferation of dermal fibroblasts and promotes their apoptosis through the chi-miR-145-5p/NUAK1 signaling axis, thereby regulating the formation of the DP structure and the morphogenesis of SHFs.

In summary, this study reveals the crucial role of lncRNA MRPS28 in the morphogenesis of SHFs, effectively regulating the proliferation and migration capabilities of dermal fibroblasts. The DP, as the “command center” for hair follicle development, is formed through continuous proliferation and differentiation of dermal fibroblasts and plays a decisive role in the normal growth and development of hair follicles [2]. We further confirm that lncRNA MRPS28, as a ceRNA for chi-miR-145-5p, can upregulate the expression of *NUAK1*, thereby indirectly affecting the development of SHFs. This discovery not only enriches the regulatory network of lncRNAs in SHF development but also provides new theoretical support and potential molecular targets for improving the quality and yield of cashmere in cashmere goats.

## 5. Conclusions

This study reveals that lncRNA MRPS28 can act as a sponge for chi-miR-145-5p, alleviating its inhibitory effect on the *NUAK1* gene, thereby regulating the proliferation and migration of dermal fibroblasts, and ultimately influencing the morphogenesis of SHFs in cashmere goats. This finding not only enriches our understanding of the regulatory network of lncRNAs in the development of SHFs but also offers a new theoretical basis and potential molecular target for enhancing the quality and yield of cashmere in cashmere goats.

## Data Availability

The raw data of Inner Mongolia cashmere goat skin transcriptome sequencing were submitted to the SRA database under accession numbers (SRR13306949, SRR13306948, SRR13306947, SRR13306946, SRR13306945, SRR13306944, SRR13306943, SRR13306942, SRR13306941, SRR13306940, SRR13306939, SRR13306938).

## References

[1] Wang M., Ma R., Ma Q., Ma B., Shang F., Lv Q., Wang Z., Wang R., Su R., Zhao Y. (2024). Role of LncRNA MSTRG.20890.1 in Hair Follicle Development of Cashmere Goats. Genes.

[2] Ma R., Du H.D., Cao Y.L., Zhao Z.Q., Shang F.Z., Wang R.J., Zhang Y.J. (2024). Research progress on the regulatory mechanisms of follicle morphogenesis and development in mammals. J. China Agric. Univ..

[3] Ma R., Shang F., Rong Y., Pan J., Wang M., Niu S., Qi Y., Li Y., Lv Q., Wang Z. (2022). Expression Profile of Long Non-Coding RNA in Inner Mongolian Cashmere Goat with Putative Roles in Hair Follicles Development. Front. Vet. Sci..

[4] Zhang Y.J., Yin J., Li J.Q., Li C.Q. (2007). Study on Hair Follicle Structure and Morphogenesis of the Inner Mongolian Arbas Cashmere Goat. Sci. Agric. Sin..

[5] Chang Y.F., Bao P.J., Chu M., Wu X.Y., Liang C.N., Yan P. (2019). Research Progress on the Regulation of lncRNA in the Development of Mammalian Hair Follicle. Biotechnol. Bull..

[6] Jiang Y., Liu H., Zou Q., Li S., Ding X. (2022). miR-29a-5p Inhibits Prenatal Hair Placode Formation Through Targeting EDAR by ceRNA Regulatory Network. Front. Cell Dev. Biol..

[7] Zhang T., Zhang Y., Li X., Zhang F., Cheng Z., Shi Y., Zhou X., Wang X. (2024). An Anti-Sense lncRNA of the A-FABP Gene Regulates the Proliferation of Hair Follicle Stem Cells via the Chi-miR-335-5p/DKK1/β-Catenin Axis. Int. J. Biol. Macromol..

[8] Zhang Y., Li F., Shi Y., Zhang T., Wang X. (2022). Comprehensive Transcriptome Analysis of Hair Follicle Morphogenesis Reveals That lncRNA-H19 Promotes Dermal Papilla Cell Proliferation through the Chi-miR-214-3p/β-Catenin Axis in Cashmere Goats. Int. J. Mol. Sci..

[9] Cao Z., Pan X., Yang Y., Huang Y., Shen H.-B. (2018). The lncLocator: A Subcellular Localization Predictor for Long Non-Coding RNAs Based on a Stacked Ensemble Classifier. Bioinformatics.

[10] Ponting C.P., Oliver P.L., Reik W. (2009). Evolution and Functions of Long Noncoding RNAs. Cell.

[11] Si Y., Bai J., Wu J., Li Q., Mo Y., Fang R., Lai W. (2018). LncRNA PlncRNA-1 Regulates Proliferation and Differentiation of Hair Follicle Stem Cells through TGF-β1-mediated Wnt/Β-catenin Signal Pathway. Mol. Med. Rep..

[12] Lin B.-J., Zhu J.-Y., Ye J., Lu S.-D., Liao M.-D., Meng X.-C., Yin G.-Q. (2020). LncRNA-XIST Promotes Dermal Papilla Induced Hair Follicle Regeneration by Targeting miR-424 to Activate Hedgehog Signaling. Cell. Signal..

[13] Wang J., Wu X., Zhang L., Wang Q., Sun X., Ji D., Li Y. (2024). miR-133a-3p and miR-145-5p Co-Promote Goat Hair Follicle Stem Cell Differentiation by Regulating NANOG and SOX9 Expression. Anim. Biosci..

[14] Link W. (2019). Introduction to FOXO Biology. Methods Mol. Biol..

[15] Ahlawat S., Arora R., Sharma R., Sharma U., Kaur M., Kumar A., Singh K.V., Singh M.K., Vijh R.K. (2020). Skin Transcriptome Profiling of Changthangi Goats Highlights the Relevance of Genes Involved in Pashmina Production. Sci. Rep..

[16] Jiao Q., Yin R.H., Zhao S.J., Wang Z.Y., Zhu Y.B., Wang W., Zheng Y.Y., Yin X.B., Guo D., Wang S.Q. (2019). Identification and Molecular Analysis of a lncRNA-HOTAIR Transcript from Secondary Hair Follicle of Cashmere Goat Reveal Integrated Regulatory Network with the Expression Regulated Potentially by Its Promoter Methylation. Gene.

[17] Gao Y., Wang X., Yan H., Zeng J., Ma S., Niu Y., Zhou G., Jiang Y., Chen Y. (2016). Comparative Transcriptome Analysis of Fetal Skin Reveals Key Genes Related to Hair Follicle Morphogenesis in Cashmere Goats. PLoS ONE.

[18] Han W., Li X., Wang L., Wang H., Yang K., Wang Z., Wang R., Su R., Liu Z., Zhao Y. (2018). Expression of Fox-Related Genes in the Skin Follicles of Inner Mongolia Cashmere Goat. Asian Australas. J. Anim. Sci..

[19] Zhang Y., Wang L., Li Z., Chen D., Han W., Wu Z., Shang F., Hai E., Wei Y., Su R. (2019). Transcriptome Profiling Reveals Transcriptional and Alternative Splicing Regulation in the Early Embryonic Development of Hair Follicles in the Cashmere Goat. Sci. Rep..

